# NDUFS8 facilitates hepatocellular carcinoma growth by enhancing mitochondrial function and escaping HUWE1-dependent degradation

**DOI:** 10.1016/j.tranon.2025.102521

**Published:** 2025-09-05

**Authors:** Xuxia Zhu, Ping Lu, Liang Ji, Qingyu Liang

**Affiliations:** Department of General Surgery, Affiliated Zhangjiagang Hospital of Soochow University

**Keywords:** HCC, Mitochondria, Apoptosis, NDUFS8,HUWE1

## Abstract

**Background:**

Hepatocellular carcinoma (HCC) is a leading cause of cancer-related mortality worldwide. Although mitochondrial metabolism contributes to tumorigenesis, the specific roles of individual mitochondrial components remain unclear.NADH:ubiquinone oxidoreductase core subunit S8 (NDUFS8), a key subunit of mitochondrial complex I, has been implicated in non-hepatic malignancies, but its functional relevance in HCC is unknown.

**Methods:**

We assessed NDUFS8 expression in HCC tissues and cell lines using TCGA datasets and patient specimens. Functional analyses—including mitochondrial assays, apoptosis, proliferation, and migration—were performed in NDUFS8-silenced, knockout, and overexpressing HCC cells. In vivo tumor growth was evaluated using xenograft mouse models. Mechanistically, mass spectrometry and immunoprecipitation identified HUWE1 as an E3 ligase responsible for NDUFS8 ubiquitination.

**Results:**

NDUFS8 was significantly overexpressed in HCC tissues and cell lines, correlating with poor patient prognosis. NDUFS8 localized to mitochondria and promoted complex I activity and ATP production. Knockdown or knockout of NDUFS8 impaired mitochondrial function, increased ROS, disrupted redox homeostasis, induced apoptosis, and suppressed proliferation and migration of HCC cells. In contrast, NDUFS8 overexpression enhanced oncogenic behaviors. In vivo, NDUFS8 silencing via AAV delivery significantly inhibited xenograft growth and triggered apoptosis. Mechanistically, HUWE1 was identified as a specific E3 ligase that ubiquitinates NDUFS8 at lysine 88, regulating its stability.

**Conclusions:**

NDUFS8 is a mitochondrial regulator that promotes HCC progression through metabolic activation and is post-translationally modified by HUWE1. Targeting NDUFS8 or its regulatory axis may represent a promising therapeutic strategy for HCC.

## Introduction

Hepatocellular carcinoma (HCC) is one of the most common malignancies worldwide and continues to pose a major challenge to global healthcare systems. While viral hepatitis and alcohol remain major risk factors, non-alcoholic fatty liver disease (NAFLD) is rapidly becoming a leading cause of HCC. The therapeutic landscape for advanced HCC has become increasingly diverse, owing to the approval of new first- and second-line agents and the establishment of immune checkpoint inhibitors as standard-of-care treatments. As a result, the prognosis for patients with advanced HCC has improved. However, the optimal sequencing of therapies remains undefined, and there is an urgent need for predictive biomarkers to guide individualized treatment selection [[1], [2], [3], [4], [5], [6]].

Mitochondria are dynamic organelles with critical metabolic functions. Beyond their roles in bioenergetics and biosynthesis, they serve as signaling hubs that coordinate key biological pathways. Emerging evidence from both human and mouse models supports the concept that active mitochondrial metabolism is essential for tumor growth. Mitochondrial activity contributes not only by supplying metabolic intermediates necessary for macromolecule biosynthesis but also by generating co-factors that sustain the cancer phenotype, thereby promoting anabolic metabolism in tumor cells [[7], [8], [9], [10]].

Expanding our understanding of the regulatory mechanisms and functional roles of mitochondria in cancer cell metabolism represents a critical avenue in biomedical research. Such insights may offer new opportunities for the development of mitochondria-targeted therapeutic strategies [[11], [12], [13]].

NADH:ubiquinone oxidoreductase core subunit S8 (NDUFS8) is a key iron–sulfur (Fe–S) cluster-containing subunit of mitochondrial complex I, directly involved in electron transport and energy metabolism [14]. Mutations in NDUFS8 have been associated with Leigh syndrome, a progressive neurodegenerative disorder of infancy linked to mitochondrial dysfunction [15,16]. Additionally, elevated serum levels of NDUFS8 have been correlated with improved insulin sensitivity in patients with type 1 diabetes [PMID: 36,135,178], and NDUFS8 has also been identified as a candidate gene for Alzheimer’s disease [17]. In the context of cancer, Hsiao et al. reported that high NDUFS8 expression is associated with poorer overall survival in non-small cell lung cancer (NSCLC) [18], while Jiang et al. demonstrated that exogenous overexpression of NDUFS8 enhances complex I activity and contributes to radioresistance in NSCLC cells [19]. Conversely, downregulation of NDUFS8 has been observed in Ming’s infiltrative gastric cancer (IGC) [20].

Despite these findings, studies on NDUFS8 in cancer remain limited. To our knowledge, our study is the first to report that NDUFS8 is upregulated in HCC and that its high expression correlates with poor patient prognosis. Furthermore, we identify the E3 ubiquitin ligase HUWE1 as a novel binding partner of NDUFS8, mediating its ubiquitination at lysine 88 (K88). These findings suggest that NDUFS8 may play a critical role in HCC pathogenesis and highlight a potential regulatory mechanism involving HUWE1-mediated post-translational modification.

## Materials and methods

### clinical specimen

#### Cells

Human cell lines LO2, SMMC-7721, Bel-7402, Huh7, HCCLM3, HepG2, and 293T were obtained from the Department of General Surgery, Affiliated Zhangjiagang Hospital of Soochow University. All cell lines underwent routine quality control assessments, including screening for mycoplasma and microbial contamination, short tandem repeat (STR) profiling for authentication, and morphological evaluation to ensure cell identity and integrity.

#### Reagents

Cell culture reagents used in this study, including fetal bovine serum (FBS), Matrigel, culture media, rotenone, antimycin A, and antibiotics, were purchased from Thermo Fisher Scientific. The anti-NDUFS8 antibody was obtained from Abcam (Cambridge, UK). Additional antibodies were kindly provided by Dr. Wang and Dr. Xu [21,22]. Polybrene and other chemical reagents were purchased from Sigma-Aldrich (St. Louis, MO, USA). Fluorescent dyes used throughout the study have been previously described [23,24].

#### JC-1

Cells were seeded into 24-well plates at a density of 1 × 10⁴ cells per well and cultured for 48 h. Mitochondrial membrane potential was assessed using JC-1 staining. Cells were incubated with JC-1 dye for 2 h according to the manufacturer’s instructions. Mitochondrial depolarization was evaluated by measuring the fluorescence intensity of the JC-1 monomer (green, 490 nm). Representative merged images of green (monomer) and red (aggregate, 625 nm) fluorescence were captured and analyzed.

#### Co-IP

Cells were cultured in 10 cm dishes until the desired confluency and subsequently lysed on ice using ice-cold immunoprecipitation (IP) buffer. The lysates were incubated with specific antibodies pre-conjugated to protein A/G agarose beads at 4 °C for 3 h with gentle rotation. Following incubation, the beads were washed three times with ice-cold IP buffer to remove non-specific bindings. The resulting immunoprecipitates, along with corresponding input controls, were subjected to either immunoblotting or mass spectrometry analysis.

#### Western blot

Cells or tissue samples were lysed on ice, and the lysates were collected using a cell scraper. The samples were then centrifuged at high speed at 4 °C to remove debris. The resulting supernatant was carefully aspirated and subjected to subsequent quantitative analyses. Detailed experimental procedures have been previously described [25].

#### Xenograft

Xenograft experiments were performed using 4- to 5-week-old male and female nude mice, which were housed in the animal facility of Soochow University under standard conditions. Each mouse was subcutaneously injected in the axillary region with 2 × 10⁶ Huh7 cells suspended in sterile PBS. Tumor growth was monitored regularly. Approximately two weeks after cell implantation, mice bearing palpable xenograft tumors were randomly assigned to two groups. Each group received intratumoral injections of the designated adeno-associated virus (AAV) at a volume of 2.5 μL per injection, with a viral titer of 1.0 × 10⁹ plaque-forming units (PFU). Injections were administered twice. All animal procedures were approved by the Institutional Animal Care and Use Committee (IACUC) and the Ethics Committee of Soochow University.

#### CCK-8

Genetically modified non-hepatocellular carcinoma cells were seeded into 96-well plates at a density of 8000 cells per well. After incubation at 37 °C with 5 % CO₂ for 48 h, 10 μL of the CCK-8 reagent (Dojindo, Japan) was added to each well. The plates were then incubated for an additional 2 h under the same conditions. Absorbance at 450 nm was measured using a microplate reader to assess cell viability.

#### Transwell migration assay

Genetically modified cells were resuspended in serum-free medium at a concentration of 1.0 × 10⁴ cells per well and seeded into the upper chamber of Transwell inserts (8.0 μm pore size, Corning, USA). After 24 h of incubation at 37 °C in a humidified incubator with 5 % CO₂, the cells that had migrated to the lower surface of the membrane were fixed with 4 % paraformaldehyde for 15 min, stained with crystal violet, and imaged under a microscope.

#### Caspase activity

Caspase-3 and caspase-9 activities were measured in cell lysates using the Caspase-3/−9 Colorimetric Assay Kit (BioVision, Milpitas, CA, USA) following the manufacturer’s protocol.

#### Cellar fluorescence staining

Cells were seeded into 12-well plates at a density of 4 × 10⁴ cells per well in 600 μL of culture medium and incubated for the indicated duration. After incubation, cells were fixed with 4 % paraformaldehyde, washed with phosphate-buffered saline (PBS), and permeabilized with 0.25 % Triton X-100 at room temperature. Subsequently, cells were incubated with the fluorescent dye, washed, and imaged using a Leica fluorescence microscope.

#### RNA extraction and qRT-PCR

Total RNA was extracted from cell or tissue lysates using TRIzol reagent (Thermo Fisher Scientific) following the manufacturer’s instructions. Complementary DNA (cDNA) was synthesized using the PrimeScript RT kit (Thermo Fisher Scientific). Quantitative real-time PCR (qRT-PCR) was subsequently performed with β-actin as the internal control. Data quantification was conducted according to previously established protocols [22]. Primers for NDUFS8 and β-actin were obtained from Genechem (Shanghai, China).

#### Lentiviral transfection and stable cell line generation

293T cells were transfected with lentiviral constructs harboring either two distinct shRNAs targeting NDUFS8 (NDUFS8-sh1 and NDUFS8-sh2) or the full-length NDUFS8 cDNA sequence, together with lentiviral envelope plasmids, using Lipofectamine 3000 (Thermo Fisher Scientific) according to the manufacturer’s protocol. Lentiviral particles were subsequently collected and used to infect hepatocellular carcinoma cells at a multiplicity of infection (MOI) of 15 for 48 h. Following infection, cells were cultured in complete medium and stably transfected cell lines were selected with puromycin. Successful NDUFS8 knockdown or overexpression was validated by both mRNA and protein analyses.

For in vivo experiments, the NDUFS8 shRNA or scramble control shRNA sequences were cloned into an adeno-associated virus (AAV) vector (from Anzhenbio,China). The resulting AAV constructs were co-transfected into 293T cells along with the AAV packaging plasmids to produce shRNA-expressing AAV particles.

#### Tissue fluorescence staining

Paraffin-embedded xenograft tissue sections were baked, dewaxed, and rehydrated following standard protocols. After washing with PBST, sections were blocked with goat serum. The primary antibody against NDUFS8 was applied and incubated overnight at 4 °C. Subsequently, sections were incubated with the fluorescent secondary antibody at room temperature for 2 h. Fluorescence images were acquired using a Zeiss microscope.

#### Oxygen consumption rate

Baseline oxygen consumption rate (OCR) was measured prior to treatment. Mitochondrial respiratory function was assessed by sequentially adding specific inhibitors and uncouplers: ATP synthase inhibitor oligomycin (Oligo) was first applied to evaluate ATP-linked respiration, followed by the addition of the oxidative phosphorylation uncoupler FCCP to determine maximal respiratory capacity. Finally, mitochondrial complex I and III inhibitors, rotenone (Rot) and antimycin A (AA), were added as negative controls to fully inhibit mitochondrial oxygen consumption, confirming assay specificity.

#### EdU staining

Genetically modified cells were seeded into 12-well plates at a density of 5 × 10⁴ cells per well and cultured for 24 h. Cell proliferation was assessed using the EdU Apollo567 kit (Invitrogen) according to the manufacturer’s instructions. Briefly, cells were incubated with EdU, followed by nuclear staining with DAPI.

#### TUNEL staining

Transgenic cells were seeded into 12-well plates at a density of 5 × 10⁴ cells per well and cultured for 48 h. Apoptosis was evaluated using the TUNEL assay kit (Invitrogen) following the manufacturer’s protocol. Cells were stained with TUNEL reagent and counterstained with DAPI, then observed under a Leica fluorescence microscope.

#### Bioinformatics analysis

The bioinformatics analysis were proficiently conducted using R 4.1, ensuring a high level of precision and reliability in the data processing and interpretation.

#### Statistical analyses

Data are presented as mean ± standard deviation (SD) and were confirmed to follow a normal distribution. All experiments were performed in triplicate. Statistical significance was defined as *p* < 0.05.

## Results

### NDUFS8 is upregulated and correlates with poor prognosis in hepatocellular carcinoma (HCC)

First, we analyzed the expression of NDUFS8 in hepatocellular carcinoma (HCC) using data from The Cancer Genome Atlas (TCGA) project. The TCGA-HCC dataset revealed that NDUFS8 transcript levels were significantly elevated in tumor tissues compared to normal liver tissues. In the paired tissue analysis, NDUFS8 expression was markedly higher in HCC tumors than in their adjacent non-tumorous counterparts. Notably, high NDUFS8 expression was significantly associated with poor clinical outcomes in HCC patients. Moreover, receiver operating characteristic (ROC) curve analysis demonstrated that NDUFS8 had strong predictive value for unfavorable survival, with an area under the curve (AUC) of 0.936, indicating high diagnostic accuracy.

Next, we identified the top 400 genes co-expressed with NDUFS8 in the TCGA-HCC cohort and visualized the 35 most strongly correlated genes in a heatmap. Subsequent Gene Ontology (GO) enrichment and Cellular Component (CC) analyses revealed that these co-expressed genes were significantly enriched in biological processes related to mitochondrial function, including ATP metabolic processes, oxidative phosphorylation, and respiratory chain complex assembly. These findings suggest that NDUFS8 may play a pivotal role in HCC by modulating mitochondrial-related pathways.

### NDUFS8 is highly expressed in HCC tissues and cell lines

To further investigate NDUFS8 expression in HCC, we analyzed tumor and adjacent normal liver tissues from 10 patients with primary HCC (*n* = 10). Quantitative PCR analysis revealed that NDUFS8 mRNA levels were significantly upregulated in tumor tissues compared to matched non-tumorous liver tissues. Western blot analysis of tumor samples from six representative patients (designated T1 to T6) showed a marked increase in NDUFS8 protein levels. A comprehensive evaluation across all ten patient samples confirmed the significant upregulation of NDUFS8 protein in HCC tumor tissues.

To determine the subcellular localization of NDUFS8, we performed immunofluorescence co-localization assays in normal liver LO2 cells and HCC Huh7 cells. NDUFS8, labeled with green fluorescence, exhibited strong co-localization with MitoTracker, a red-fluorescent mitochondrial marker, indicating mitochondrial localization of NDUFS8. Notably, the fluorescence intensity of NDUFS8 was markedly higher in Huh7 cells than in LO2 cells.

In addition, we assessed NDUFS8 expression across multiple HCC cell lines. qRT-PCR analysis revealed that NDUFS8 mRNA levels were significantly elevated in SMMC-7721, Bel-7402, Huh7, HCCLM3, and HepG2 cells compared to the LO2 normal liver cell line. This upregulation was further validated at the protein level by Western blotting, which consistently showed higher NDUFS8 protein expression in all tested HCC cell lines, while its expression remained low in normal liver cells.

Collectively, these results demonstrate that NDUFS8 is significantly upregulated in HCC tissues and cell lines and is predominantly localized to mitochondria, suggesting a potential role in HCC tumorigenesis and progression.

Fig. 1, Fig. 2

**Fig. 1:**
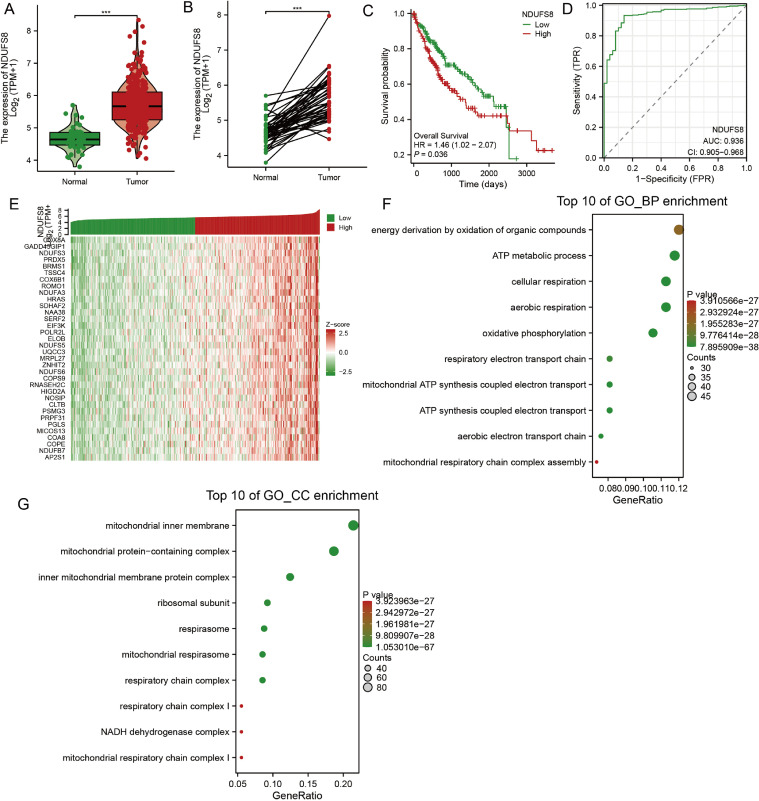
mRNA expression levels of NDUFS8 in HCC tumour tissue (‘Tumour’), normal liver tissue (‘Normal’) (A), and matched peritumoral normal tissue (B) in the TCGA hepatocellular carcinoma (HCC) cohort. Kaplan-Meier survival curve analysis shows the correlation between NDUFS8 expression and overall survival in HCC patients (C). Receiver operating characteristic (ROC) curve analysis evaluates the predictive value of NDUFS8 overexpression for HCC patient survival, showing true positive rate (TPR) and false positive rate (FPR) (D). Gene heatmap showing the top 35 genes most highly correlated with NDUFS8 expression in the TCGA HCC cohort. GO enrichment analysis was performed using the top 400 genes most highly correlated with NDUFS8 expression, and the top 10 enriched terms in Gene Ontology (GO) and Cellular Component (CC) are shown. ***p < 0.001.

**Fig. 2:**
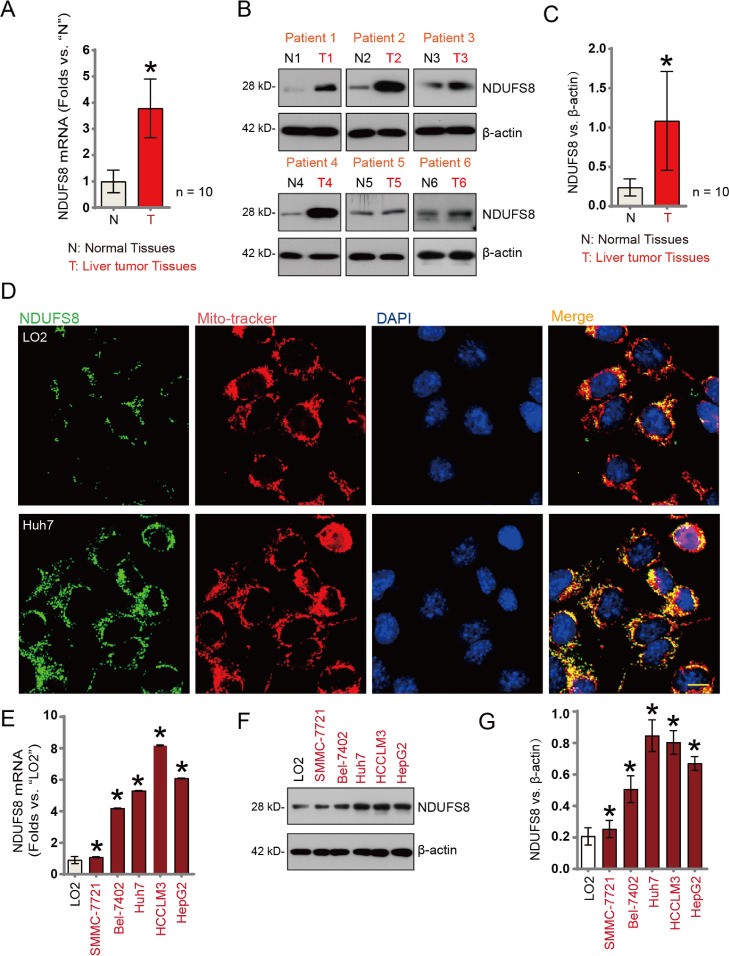
mRNA and protein expression levels of NDUFS8 in tumour tissue (‘T’) and matched adjacent normal liver tissue (‘N’) from 10 HCC patients (*n* = 10) (A-C). Immunofluorescence staining of tissues shows the subcellular localisation of NDUFS8 (green), mitochondrial dye MitoTracker (red), and nuclear stain DAPI (blue) in tumour and adjacent normal tissues. mRNA (E) and protein expression (F, G) of NDUFS8 in HCC cell lines and normal liver cell lines (LO2). Error bars represent mean ± standard deviation (SD). Differences are statistically significant compared to ‘Normal’ tissue or ‘LO2’ cells. All experiments were independently repeated three times. *p < 0.05. Scale bar = 50 μm.

### Silencing of NDUFS8 impairs mitochondrial function in hepatocellular carcinoma cells

**Fig. 3:**
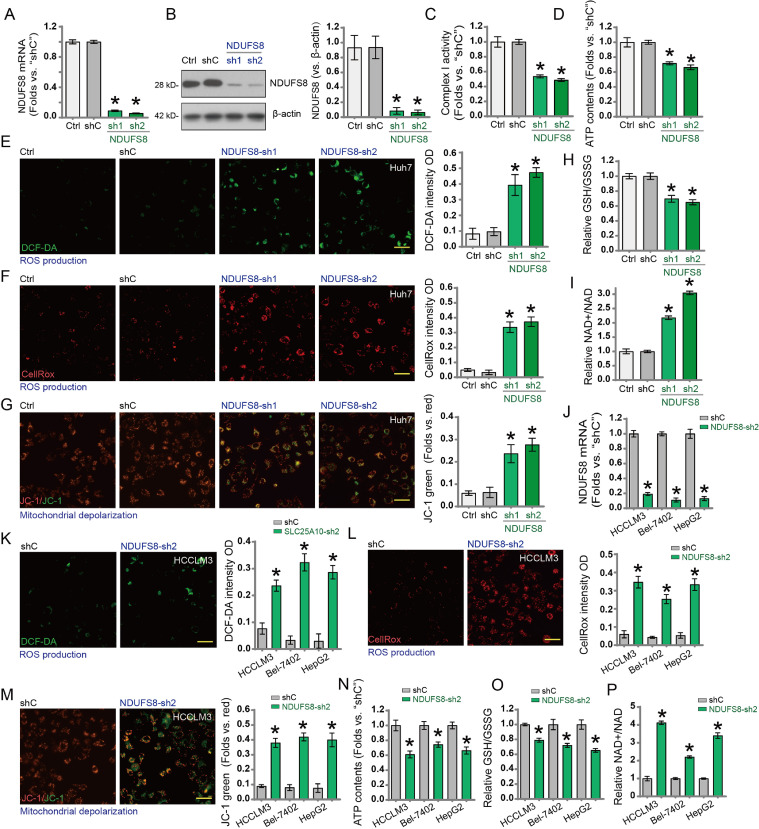
HCC cells stably expressing lentiviral vector-mediated NDUFS8-targeting shRNA (NDUFS8-sh1 or NDUFS8-sh2) or control shRNA (shC). NDUFS8 mRNA (A) and protein expression (B) were detected after 24 h of culture. Mitochondrial complex I activity (C), cellular ATP content (D), ROS production (DCF-DA and Cell Rox dyes, E, F), and mitochondrial depolarisation (JC-1 monomer intensity, G) were measured. The GSH/GSSG ratio (H) and NAD+/NADH ratio (I) were determined. Additionally, after stable expression of NDUFS8-sh2 or shC in HCCLM3, Bel-7402, and HepG2 cells, NDUFS8 mRNA (J), ROS production (K, L), mitochondrial depolarisation (M), ATP content (N), GSH/GSSG (O), and NAD+/NAD (P) ratios were measured after 24 h of culture. ‘Ctrl’ represents wild-type cells. Error bars represent mean ± standard deviation (SD). Differences were significant compared to the “shC” group. *p < 0.05. All experiments were independently repeated three times. Scale bar = 100 μm.

To investigate the potential function of NDUFS8 in hepatocellular carcinoma (HCC) cells, we silenced NDUFS8 using two non-overlapping shRNA sequences (NDUFS8-sh1 and NDUFS8-sh2). Compared to Huh7 cells expressing control scramble shRNA (shC), NDUFS8 mRNA (Fig. 3A) and protein levels (Fig. 3B) were significantly reduced in cells expressing NDUFS8 shRNA. Silencing NDUFS8 markedly impaired mitochondrial function in Huh7 cells, as evidenced by decreased mitochondrial complex I activity (Fig. 3C) and reduced intracellular ATP levels (Fig. 3D). Additionally, reactive oxygen species (ROS) levels were significantly increased in NDUFS8-silenced cells, as indicated by enhanced DCF-DA green fluorescence (Fig. 3E) and CellROX red fluorescence intensity. Changes in mitochondrial membrane potential were further confirmed by JC-1 staining, with a reduction in red aggregates and a notable increase in green monomers in the silenced group, indicating mitochondrial depolarization (Fig. 3G). Moreover, silencing NDUFS8 significantly reduced the GSH/GSSG ratio (Fig. 3H) and the NAD⁺/NADH ratio, further demonstrating mitochondrial dysfunction accompanied by enhanced oxidative stress.

**Fig. 4:**
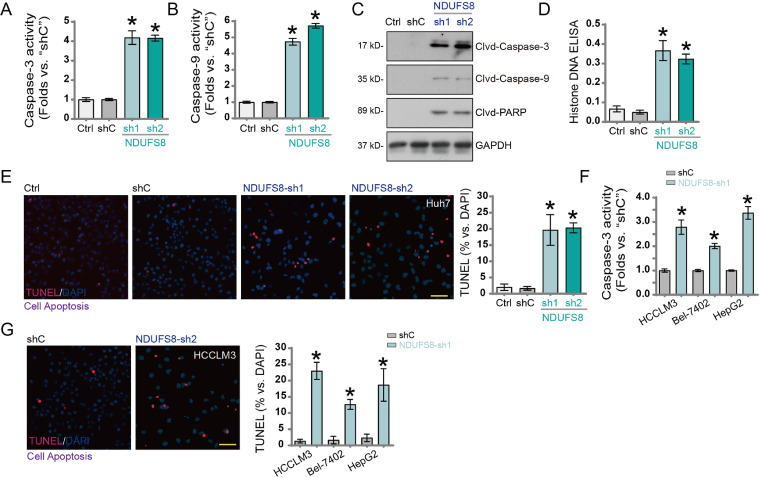
Detection of Caspase-3 (A) and Caspase-9 (B) activity, apoptosis-related protein expression (C), and histone-bound DNA content (D) in cultured cells after 72 h. Apoptosis was detected and quantified via nuclear TUNEL staining (E). Caspase-3 activity (F) and TUNEL staining (G) were detected in HCCLM3, Bel-7402, and HepG2 cells transfected with NDUFS8-sh1. ‘Ctrl’ represents wild-type cells. Error bars indicate mean ± standard deviation (SD). Significant differences were observed compared to the ‘shC’ group. *p < 0.05. All experiments were independently repeated three times. Scale bar = 100 μm.

To verify the role of NDUFS8 in other HCC cell lines, we transduced NDUFS8-sh2 into HCCLM3, Bel-7402, and HepG2 cells using lentiviral vectors and established stable cell lines via puromycin selection. Compared to the shC control group, NDUFS8 mRNA expression was significantly downregulated in NDUFS8-sh2 cells (Fig. 4K). Concurrently, DCF-DA and CellROX fluorescence intensities increased, indicating elevated ROS levels. JC-1 staining revealed prominent mitochondrial depolarization and a significant decline in ATP content (Fig. 4L). Furthermore, reductions in GSH/GSSG and NAD⁺/NADH ratios supported the oxidative redox imbalance induced by NDUFS8 silencing.

Collectively, these results indicate that NDUFS8 silencing disrupts mitochondrial function and cellular redox homeostasis, significantly affecting the metabolic state and physiological functions of HCC cells.

### NDUFS8 silencing induces apoptosis in hepatocellular carcinoma cells

Given the critical role of mitochondrial function in apoptosis (PMID: 33,785,842), we hypothesized that NDUFS8 silencing may regulate apoptotic processes in HCC cells. In Huh7 cells, silencing NDUFS8 using NDUFS8-sh1 and NDUFS8-sh2 significantly increased Caspase-3 (Fig. 4A) and Caspase-9 (Fig. 4B) activity. Western blot analysis showed that NDUFS8 silencing induced cleavage of Caspase-3, Caspase-9, and poly(ADP-ribose) polymerase 1 (PARP1) (Fig. 4C). ELISA results showed a significant increase in histone-bound DNA fragments in NDUFS8-silenced Huh7 cells (Fig. 4D), indicating increased DNA fragmentation. TUNEL staining further confirmed that NDUFS8 silencing markedly increased the proportion of TUNEL-positive nuclei in Huh7 cells (Fig. 4E), indicating apoptosis activation.

Similarly, in other immortalized HCC cell lines including HCCLM3, Bel-7402, and HepG2, NDUFS8-sh1-mediated silencing significantly increased Caspase-3 activity (Fig. 4F). Correspondingly, the proportion of TUNEL-positive nuclei also increased (Fig. 4G), further supporting apoptosis induction upon NDUFS8 silencing.

### NDUFS8 silencing suppresses malignant biological behaviors of HCC cells

**Fig. 5:**
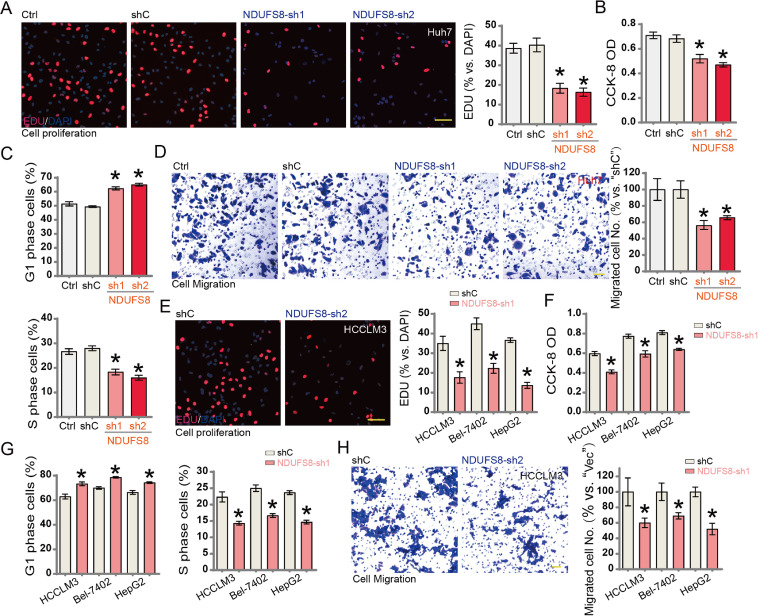
After the specified culture time, cell cycle progression was analysed by EdU staining of cell nuclei (A, E), CCK-8 assay for cell viability (B, F), and PI-FACS analysis (C, G). Cell migration ability was assessed by Transwell migration assay (D, H). ‘Ctrl’ represents wild-type cells. Error bars indicate mean ± standard deviation (SD). Compared with the ‘shC’ group, the differences were significant. *p < 0.05. All experiments were independently repeated three times. Scale bar = 100 μm.

Excessive mitochondrial activity has been identified as a key driver of hepatocellular carcinoma (HCC) progression [[26], [27], [28]]. Accordingly, we assessed the impact of NDUFS8 silencing on HCC cell progression in vitro. Silencing NDUFS8 with NDUFS8-sh1 and NDUFS8-sh2 in Huh7 cells significantly suppressed cell proliferation, as shown by reduced EdU-positive nuclei (Fig. 5A). CCK-8 assay revealed a notable reduction in cell viability (Fig. 5B). Moreover, NDUFS8 silencing induced cell cycle disruption, causing G1 phase arrest, indicated by an increased proportion of G1 phase cells and a decrease in S phase cells (Fig. 5C). Functional analysis demonstrated that NDUFS8 shRNA significantly inhibited the migratory capacity of Huh7 cells in vitro (Fig. 5D).

Similarly, in HCCLM3, Bel-7402, and HepG2 cell lines, NDUFS8-sh2-mediated silencing reduced EdU-positive nuclei (Fig. 5E) and cell proliferation (Fig. 5F). NDUFS8 silencing also induced cell cycle arrest (Fig. 5G) and inhibited migration (Fig. 5H), suggesting a crucial role for NDUFS8 in regulating proliferation and migration of HCC cells, and highlighting its potential as a therapeutic target.

### NDUFS8 knockout exhibits strong anti-HCC activity

**Fig. 6:**
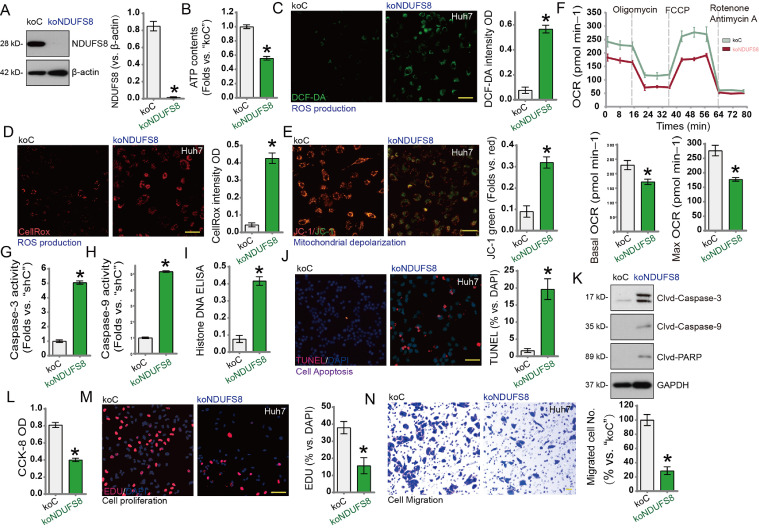
Huh7 cells stably expressing the Cas9 construct and CRISPR/Cas9-mediated NDUFS8 knockout (‘koNDUFS8’), with control cells expressing Cas9 and the control construct (‘koC’). NDUFS8 protein expression (A), ATP content (B), ROS production (DCF-DA and Cell Rox dyes, C, D), mitochondrial depolarisation (JC-1 monomer intensity, E), and cellular oxygen consumption rate (Seahorse analysis, F) were detected at specified time points. Caspase-3 (G), Caspase-9 (H) activity, and DNA-binding histone content (I) were measured, along with nuclear TUNEL apoptosis detection (J) and apoptosis-related protein expression (K). Cell viability and proliferation were assessed using CCK-8 (L) and EdU staining (M), and cell migration was evaluated via Transwell assay (N). ‘koC’ served as the control group. Error bars indicate mean ± standard deviation (SD). Differences compared with ‘koC’ were statistically significant. *p < 0.05. All experiments were independently repeated three times. Scale bar = 100 μm.

To further validate the role of NDUFS8 in HCC cells, we used CRISPR/Cas9 technology to knock out the NDUFS8 gene. A lentiviral CRISPR/Cas9 vector carrying a specific sgRNA targeting NDUFS8 was constructed and transduced into Huh7 cells expressing Cas9. After puromycin selection and validation of knockout efficiency, a monoclonal NDUFS8 knockout cell line (koNDUFS8) was established. Compared to control cells expressing non-targeting sgRNA (koC), NDUFS8 protein expression and ATP levels were significantly reduced in koNDUFS8 cells (Figs. 6A and 6B). Additionally, ROS production was increased, as evidenced by elevated green (DCF-DA) and red (CellROX) fluorescence (Figs. 6C and 6D).

NDUFS8 knockout further impaired mitochondrial function, as shown by mitochondrial depolarization and increased JC-1 green monomers (Fig. 6E). Oxygen consumption rate (OCR) assays revealed reduced basal respiration and maximal respiratory capacity in koNDUFS8 cells, indicating suppressed mitochondrial oxidative metabolism (Fig. 6F). Functionally, Caspase-3 and Caspase-9 activities were significantly increased (Figs. 6G and 6H), DNA fragmentation was elevated (Fig. 6I), and the proportion of TUNEL-positive nuclei was markedly higher in koNDUFS8 cells (Fig. 6J), all indicating enhanced apoptosis. Western blot analysis confirmed increased cleavage of Caspase-3, Caspase-9, and PARP1 in koNDUFS8 cells (Fig. 6K), supporting apoptosis activation.

Functional assays showed that NDUFS8 knockout significantly reduced cell proliferation, as demonstrated by decreased CCK-8 OD values (Fig. 6L) and lower EdU incorporation (Fig. 6M). In addition, the migration capacity of koNDUFS8 cells was significantly suppressed (Fig. 6N). In summary, CRISPR/Cas9-mediated NDUFS8 knockout induced mitochondrial dysfunction and oxidative stress in HCC cells, promoted apoptosis, and inhibited proliferation and migration, further confirming the critical role of NDUFS8 in HCC progression.

### NDUFS8 overexpression exerts oncogenic effects in HCC cells

**Fig. 7:**
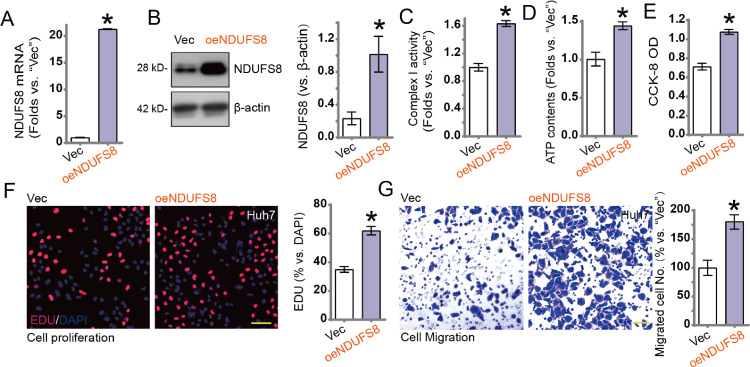
Huh7 cells stably expressing lentiviral vector-mediated NDUFS8 overexpression constructs (‘oeNDUFS8’), with control cells expressing an empty vector (‘Vec’). NDUFS8 mRNA (A) and protein (B) expression were measured at designated time points. Mitochondrial complex I activity (C), ATP content (D), cell viability assessed by CCK-8 assay (E), cell proliferation evaluated by EdU staining (F), and Transwell migration (G) were measured. Error bars indicate mean ± standard deviation (SD), with significant differences compared to the ‘Vec’ group. *p < 0.05. All experiments were independently repeated three times. Scale bar = 100 μm.

Given the potential oncogenic role of NDUFS8, we established a stable Huh7 cell line with exogenous overexpression of NDUFS8. Lentiviral vectors carrying NDUFS8 overexpression sequences were transduced into Huh7 cells, and stable cell lines (oeNDUFS8) were selected using puromycin. Compared with vector control (Vec) cells, NDUFS8 mRNA (Fig. 7A) and protein levels (Fig. 7B) were significantly upregulated in oeNDUFS8 cells. Functional assays showed that NDUFS8 overexpression significantly enhanced mitochondrial complex I activity (Fig. 7C) and intracellular ATP levels (Fig. 7D).

Moreover, NDUFS8 overexpression promoted proliferation in pNSCLC1 cells, as shown by increased OD values in CCK-8 assays (Fig. 7E) and elevated EdU-positive nuclei (Fig. 7F). Transwell migration assays further demonstrated that NDUFS8 overexpression significantly enhanced the migratory capacity of Huh7 cells.

### HUWE1 specifically mediates ubiquitination of NDUFS8 at lysine 88

**Fig. 8:**
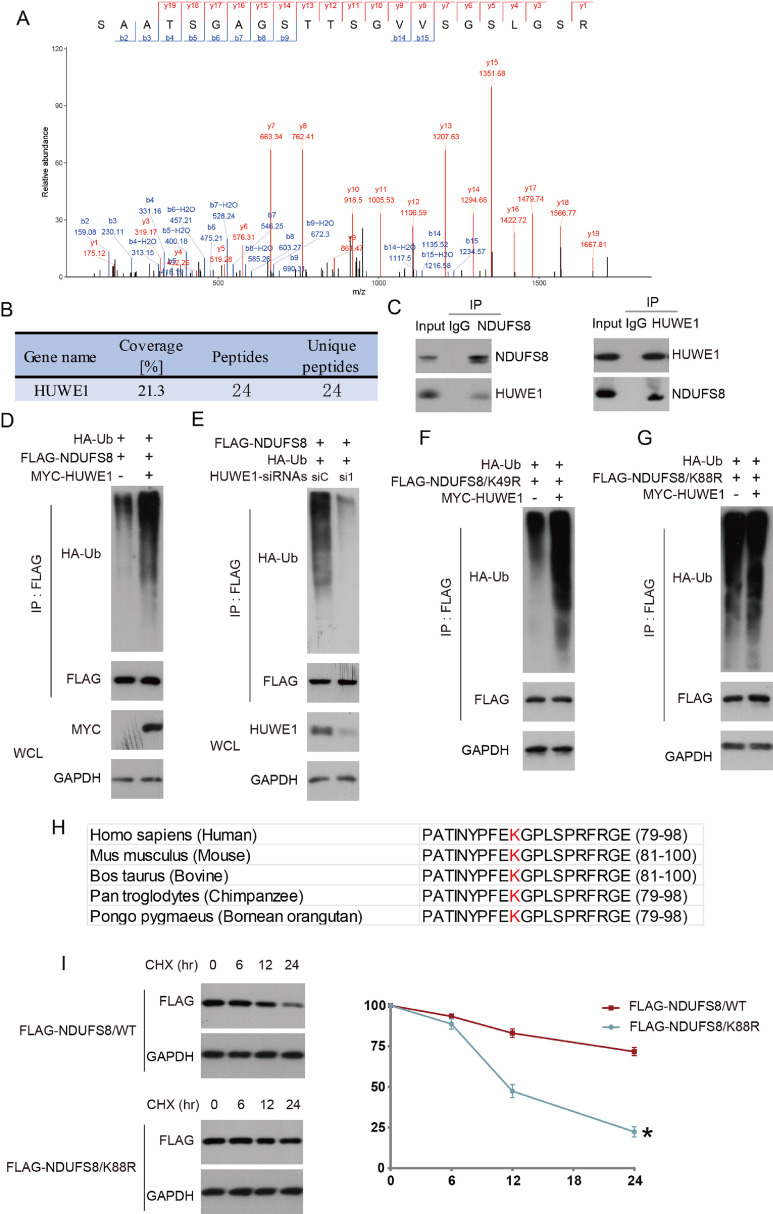
Identification of NDUFS8 interacting proteins by mass spectrometry, showing the secondary mass spectrum of the HUWE1 protein (A) and related information (B). Immunoprecipitation (Co-IP) validated the interaction between NDUFS8 and HUWE1 in Huh7 cells (C). Huh7 cells were transfected with HA-tagged ubiquitin plasmid (HA-Ub), FLAG-tagged NDUFS8 plasmid (FLAG-NDUFS8), MYC-tagged HUWE1 plasmid (MYC—HUWE1), HUWE1 siRNA (si1), or NDUFS8 key lysine site mutant plasmids (FLAG-NDUFS8/K49R, FLAG-NDUFS8/K88R), and the ubiquitination levels of the corresponding proteins were detected (D–G). The conserved sequences of the NDUFS8 protein in different species are shown (H). Detection and quantification of the expression of the relevant proteins in cells transfected with NDUFS8 wild-type (FLAG-NDUFS8/WT) and K88R mutant (FLAG-NDUFS8/K88R) (I). Error bars indicate the mean ± standard deviation (SD), and differences compared with the ‘FLAG-NDUFS8/WT’ group are significant. *p < 0.05. All experiments were independently repeated three times.

To elucidate the molecular mechanism by which NDUFS8 regulates HCC progression, we performed immunoprecipitation (pull-down) using Flag-tagged NDUFS8 in 293T cells and identified interacting proteins by mass spectrometry. The E3 ubiquitin ligase HUWE1 emerged as the top candidate based on unique peptide counts. MS/MS spectra and related identification details of HUWE1 are shown in Figs. 8A and 8B

As a key enzyme in the ubiquitin-proteasome system (UPS), E3 ligases recognize specific substrates and catalyze their ubiquitination. Ubiquitination, a crucial post-translational modification, regulates protein degradation, function, localization, and interactions [[29], [30], [31], [32], [33]]. HUWE1, a multifunctional HECT-type E3 ligase, catalyzes monoubiquitination and formation of K6-, K48-, and K63-linked polyubiquitin chains, targeting substrates critical for cellular development and homeostasis [34,35]. Previous studies implicate HUWE1 in inflammasome assembly [36], hepatic iron metabolism [37], and tumor progression [38,39], but its role in HCC remains unclear.

Based on the mass spectrometry data, we performed endogenous co-immunoprecipitation (Co-IP) in Huh7 cells and confirmed the interaction between NDUFS8 and HUWE1 (Fig. 8C). Considering HUWE1’s E3 ligase activity, we hypothesized that it may regulate NDUFS8 via ubiquitination. To test this, we co-transfected Huh7 cells with Flag-NDUFS8, Myc-HUWE1, and HA-tagged ubiquitin (HA-Ub). HUWE1 overexpression markedly increased NDUFS8 ubiquitination (Fig. 8D). Conversely, knockdown of HUWE1 with siRNA significantly reduced NDUFS8 ubiquitination (Fig. 8E), confirming HUWE1’s specific role in this process.

Using the PhosphoSite database, K49 and K88 were identified as the top two potential ubiquitination sites. Mutation of K49 to arginine (K49R) did not prevent HUWE1-mediated ubiquitination (Fig. 8F), whereas K88 mutation (K88R) abolished this effect (Fig. 8G), indicating that K88 is the specific ubiquitination site mediated by HUWE1. K88 is highly conserved across species (Fig. 8H), supporting its functional importance. Finally, comparison of NDUFS8 expression in Huh7 cells transfected with either wild-type or K88R mutant NDUFS8 revealed that K88 is critical for NDUFS8 ubiquitination and protein stability (Fig. 8I).

### NDUFS8 silencing inhibits growth of HCC xenografts in nude mice

**Fig. 9:**
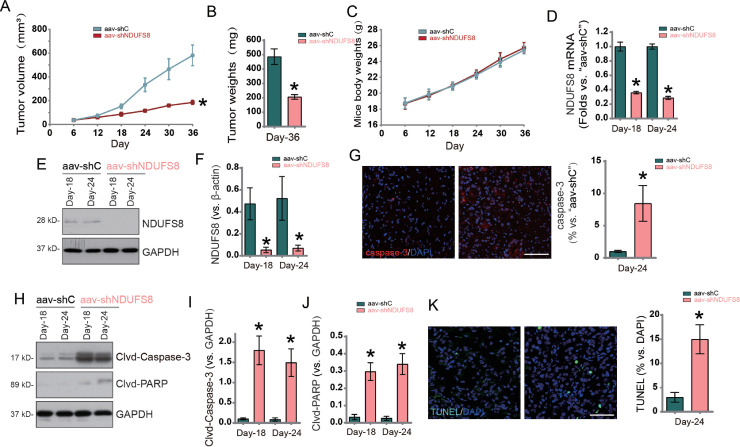
After subcutaneous transplantation of Huh7 xenografts into nude mice, intratumoral injections of adeno-associated virus (AAV) carrying NDUFS8-specific shRNA (‘AAV-shNDUFS8’) or control shRNA (‘AAV-shc’) were administered in two rounds, with a 48-hour interval between rounds. Tumour volume (A) and mouse body weight (C) were monitored during the 36-day experimental period (Day 0 to Day 36). On Day 36, all xenografts were excised and weighed (B). One xenograft was randomly selected from each group to detect NDUFS8 and related gene mRNA (D) and protein expression (E, F, H, I, J). Tumour sections were analysed by immunofluorescence for Caspase-3 (G) and TUNEL staining (K). Error bars indicate mean ± standard deviation (SD). Differences compared with the ‘AAV-shc’ group were statistically significant. *p < 0.05. *n* = 5 per group. Scale bar = 100 μm.

To evaluate the in vivo role of NDUFS8 in HCC cell growth, we established a subcutaneous xenograft model in nude mice using Huh7 cells. Two million Huh7 cells were injected subcutaneously into the axilla of each mouse, and tumors became palpable after three weeks (defined as "Day 0″). The experimental group received intratumoral injections of AAV carrying NDUFS8 shRNA (AAV-shNDUFS8), while the control group received AAV carrying scramble shRNA (AAV-shC). Injections were repeated every 48 h for two cycles. Tumor growth curves showed that AAV-shNDUFS8 significantly inhibited tumor growth, with tumor volumes markedly smaller than those in the control group (Fig. 9A). On day 36, tumors were harvested and weighed, and the AAV-shNDUFS8 group had significantly lower tumor weights (Fig. 9B). No significant differences in body weight were observed between groups, indicating good treatment safety (Fig. 9C).

To explore signaling pathway alterations, xenograft tissues were collected on days 18 and 24 for analysis. NDUFS8 mRNA and protein levels were significantly downregulated in the AAV-shNDUFS8 group (Figs. 9D and 9E). Immunofluorescence revealed an increase in caspase-3-positive cells (red fluorescence) in the AAV-shNDUFS8 group (Fig. 9G). Western blot analysis demonstrated increased cleavage of Caspase-3 and PARP1 in tumors from the AAV-shNDUFS8 group (Figs. 9H–9J). TUNEL staining showed a higher proportion of apoptotic nuclei in the xenografts following NDUFS8 silencing (Fig. 9K).

In conclusion, in vivo gene silencing of NDUFS8 via AAV delivery significantly inhibited xenograft tumor growth and induced apoptosis, supporting NDUFS8 as a critical promoter of HCC progression.

## Discussion

Building upon our comprehensive in vitro and in vivo findings, we discuss the implications of NDUFS8 dysregulation in HCC and its potential as a therapeutic target.Hepatocellular carcinoma (HCC) is the fourth leading cause of cancer-related death worldwide, with HCC being the most common subtype of liver cancer. Despite some progress in systemic treatments such as molecular targeted therapies, HCC remains one of the most lethal malignancies due to drug resistance, frequent recurrence, and metastasis [[40], [41], [42], [43]]. Therefore, there is an urgent need to identify novel and effective molecular targets for therapy. Currently, studies on the expression and function of NDUFS8 in tumors are relatively limited. Hsiao et al. reported that NDUFS8 and NDUFS1, which reflect the metabolic state of tumors, could serve as potential prognostic indicators in lung cancer [18]. Cheng et al. demonstrated that upregulation of the mitochondrial iron-sulfur protein NDUFS8 promotes tumor cell proliferation, survival, migration, and epithelial-mesenchymal transition (EMT) by increasing mitochondrial reactive oxygen species (ROS) and activating MAPK and Ras-ERK signaling pathways [44].

This study further validated the critical role of NDUFS8 in maintaining mitochondrial function and promoting HCC progression. In multiple immortalized HCC cell lines, silencing or knocking out NDUFS8 significantly impaired mitochondrial function, as evidenced by decreased mitochondrial complex I activity, ATP depletion, mitochondrial membrane depolarization, elevated ROS levels, and reduced oxygen consumption rate (OCR). In contrast, exogenous overexpression of NDUFS8 enhanced complex I activity and ATP production. These findings suggest that NDUFS8 drives HCC cell proliferation and progression by sustaining hyperactive mitochondrial function.

Based on TCGA database analyses, NDUFS8 is consistently overexpressed in HCC tissues and strongly correlates with poor patient prognosis. We observed widespread upregulation of NDUFS8 expression in multiple human HCC tissue samples and cell lines. Functional experiments showed that silencing or knockout of NDUFS8 induced apoptosis and significantly suppressed cell viability, proliferation, and migration. Conversely, exogenous overexpression of NDUFS8 promoted HCC cell proliferation and motility. in vivo studies, intratumoral injection of an adeno-associated virus carrying NDUFS8-targeting shRNA (AAV-shNDUFS8) significantly inhibited the growth of subcutaneous HCC xenografts in nude mice, further supporting the therapeutic potential of targeting NDUFS8.

Moreover, we identified HUWE1 as a specific E3 ubiquitin ligase for NDUFS8 and found that increased HUWE1 expression promotes the ubiquitination and degradation of NDUFS8, affecting its protein stability. Site-directed mutagenesis revealed that lysine 88 (K88), which is highly conserved across species, is a critical site for HUWE1-mediated ubiquitination of NDUFS8.

It is worth noting that no specific inhibitors targeting NDUFS8 have been developed so far. The feasibility and underlying mechanisms of targeting NDUFS8 as a drug target require further investigation.

## Ethics statement

This study was approved by Ethics Committee of Soochow University.

## Data availability

All data are available upon request.

## CRediT authorship contribution statement

**Xuxia Zhu:** Formal analysis, Data curation, Conceptualization. **Ping Lu:** Methodology, Investigation. **Liang Ji:** Formal analysis, Data curation. **Qingyu Liang:** Writing – review & editing, Writing – original draft, Resources, Methodology.

## Declaration of competing interest

The authors have declared that no competing interest exists.

## Appendix Supplementary materials

Image, image 1
