# Supplementary figures and images for: NDUFS8 facilitates hepatocellular carcinoma growth by enhancing mitochondrial function and escaping HUWE1-dependent degradation
Source: Transl Oncol. 2025 Sep 5;61:102521. doi: 10.1016/j.tranon.2025.102521 (PMC12447896; doi:10.1016/j.tranon.2025.102521)

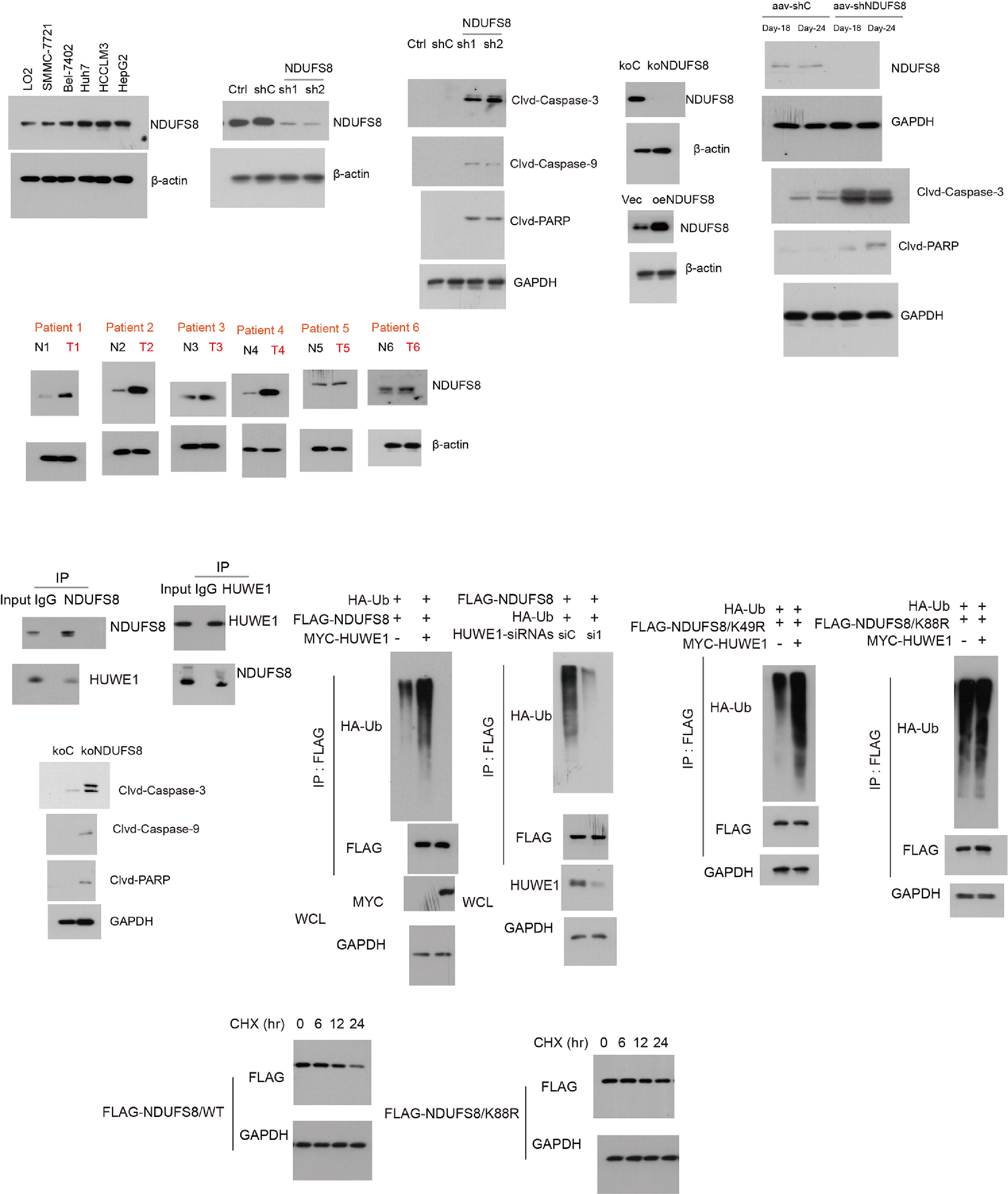

Supplement: Supplementary file 1 [file mmc1.jpg]
